# Special Section Guest Editorial: Computational Approaches for Neuroimaging

**DOI:** 10.1117/1.NPh.9.4.041401

**Published:** 2022-09-02

**Authors:** Lei Tian, Xavier Intes, Weijian Yang

**Affiliations:** a Boston University, Department of Electrical and Computer Engineering, Boston, Massachusetts, United States; b Boston University, Department of Biomedical Engineering, Boston, Massachusetts, United States; c Rensselaer Polytechnic Institute, Department of Biomedical Engineering, Troy, New York, United States; d University of California, Davis (UC Davis), Department of Electrical and Computer Engineering, Davis, California, United States

## Abstract

This guest editorial provides an introduction to the Special Section on Computational Approaches for Neuroimaging.

New advances in computational methods are revolutionizing our ability to collect, reconstruct, analyze, and interpret neuroimaging data. In turn, they provide unique new approaches to understand brain activities and functions at subcellular, cellular, circuit and network levels. This special section collates ten contributions that cover many recent computational technologies underlying these developments. These computational techniques address several challenges, including recording of neural activities, reconstruction of neural activities, inference of functional connectivity, and interpretation of the brain function at cellular level and circuit level. This special section of *Neurophotonics* Volume 9 Issue 4 consists of five review articles and five research papers. These contributions are representative of the broad range of optical technologies employed in neuroscience, such as one-photon or multi-photon imaging of functional indicators, e.g., calcium, voltage, and neurotransmitter, functional near-infrared spectroscopies to image hemodynamics, and optical intrinsic signal imaging. Beyond imaging, this special section also covers the rising field of photostimulation.

A major focus of emerging computational approaches is towards fast and accurate data processing for functional neuroimaging data. Benisty et al.^1^ provide a comprehensive review on data processing of functional optical microscopy for neuroscience, which surveys a broad range of techniques to handle massive spatiotemporal datasets from fluorescent microscopes in order to uncover neuronal activity related to behavior and stimuli, and local circuits in the brain. Cai et al.^2^ provide a focused review on data analysis methods for mesoscale neural imaging *in vivo*, which is timely since mesoscale imaging at high resolution has become one of the main frontier in neuroimaging. Carrillo-Reid and Calderon^3^ present a review on conceptual framework for neuronal ensemble identification and manipulation related to behavior using calcium imaging, which discusses computational approaches to infer neuronal ensembles from calcium imaging in behaving mice. Eastmond et al.^4^ offer a comprehensive review on deep learning in functional near-infrared spectroscopy (fNIRS), which covers the many applications of deep learning in fNIRS for brain-computer interface, neuro-impairment diagnosis, and neuroscience discovery. Gao et al.^5^ report a novel denoising autoencoder deep learning model to remove motion artifacts that plague fNIRS data in real world applications. White et al.^6^ compare data processing methods for optical intrinsic signal imaging to analyze resting-state functional connectivity in mice by processing temporal and spatial autocorrelations. Dehkharghanian et al.^7^ present a new semi-automated machine learning algorithm for analyzing bioluminescence images in order to measure adenosine triphosphate (ATP) indicators in neurons.

Another emerging area is computational imaging, which seeks to synergistically combine novel optical designs and advanced computational algorithms to enable novel capabilities. Xue^8^ provides a review that summarizes recent advances in computational optics for high-throughput imaging of neural activity, which aims to achieve 3D parallelized excitation and detection across millimeter-scale field-of-view with micron-scale resolution at high speeds. Eybposh et al.^9^ (issue cover) provide a focused review on advances in computer-generated holography (CGH) for targeted neuronal modulation, which highlights novel algorithms and hardware implementations of advanced CGH techniques to shape the light intensity distribution in 3D for optically interrogating neuronal populations in conjugation with optogenetics. Howe et al.^10^ assessed a popular computational imaging technique, known as light field microscopy, for the extraction of neuronal calcium transients in 3D from a single-shot measurement.

In summary, computational approaches will continue to play a central role in advancing neuroimaging. Driven by the need to handle massive spatiotemporal data, we expect fast and accurate data processing pipelines that can leverage advanced deep learning frameworks will be a trend in the coming years. In order to overcome physical limitations to achieve large field-of-view, high spatial resolution, ultrafast acquisition speed, deep penetration, high signal-to-noise, and system simplicity, we expect computational optics will continue to advance and push the boundaries of neural imaging technologies and subsequently, our understanding of the brain at work.
